# *N*′-[1-(2-Hy­droxy­phen­yl)ethyl­idene]thio­phene-2-carbohydrazide

**DOI:** 10.1107/S1600536810050002

**Published:** 2010-12-04

**Authors:** Jin-He Jiang

**Affiliations:** a Microscale Science Institute, Department of Chemistry and Chemical Engineering, Weifang University, Weifang 261061, People’s Republic of China

## Abstract

The title compound, C_13_H_12_N_2_O_2_S, was prepared by the reaction of 1-(2-hy­droxy­phen­yl)ethanone and thio­phene-2-carbohydrazide. The dihedral angle between the benzene and thio­phene rings is 10.07 (17)°. An intra­molecular O—H⋯N hydrogen bond may influence the mol­ecular conformation. In the crystal, mol­ecules are linked by N—H⋯O hydrogen bonds into chains along [010].

## Related literature

For applications of Schiff base compounds, see: Casas *et al.* (2000 ▶); Habermehl *et al.* (2006 ▶). For related structures, see: Li & Jian (2010 ▶); Li & Meng (2010 ▶).
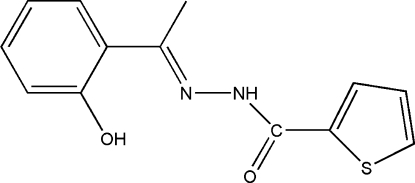


## Experimental

### 

#### Crystal data

C_13_H_12_N_2_O_2_S
*M* *_r_* = 260.31
Orthorhombic, 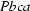

*a* = 13.454 (3) Å
*b* = 7.6303 (15) Å
*c* = 24.305 (5) Å
*V* = 2495.1 (9) Å^3^
*Z* = 8
Mo *K*α radiation
μ = 0.25 mm^−1^
*T* = 293 K
0.25 × 0.20 × 0.19 mm

#### Data collection

Bruker SMART CCD diffractometer
16044 measured reflections
2189 independent reflections
1047 reflections with *I* > 2σ(*I*)
*R*_int_ = 0.156

#### Refinement

*R*[*F* ^2^ > 2σ(*F* ^2^)] = 0.050
*wR*(*F* ^2^) = 0.126
*S* = 0.89
2189 reflections
172 parameters
H atoms treated by a mixture of independent and constrained refinement
Δρ_max_ = 0.23 e Å^−3^
Δρ_min_ = −0.29 e Å^−3^

### 

Data collection: *SMART* (Bruker, 1997 ▶); cell refinement: *SAINT* (Bruker, 1997 ▶); data reduction: *SAINT*; program(s) used to solve structure: *SHELXS97* (Sheldrick, 2008 ▶); program(s) used to refine structure: *SHELXL97* (Sheldrick, 2008 ▶); molecular graphics: *SHELXTL* (Sheldrick, 2008 ▶); software used to prepare material for publication: *SHELXTL*.

## Supplementary Material

Crystal structure: contains datablocks global, I. DOI: 10.1107/S1600536810050002/lh5171sup1.cif

Structure factors: contains datablocks I. DOI: 10.1107/S1600536810050002/lh5171Isup2.hkl

Additional supplementary materials:  crystallographic information; 3D view; checkCIF report

## Figures and Tables

**Table 1:** Hydrogen-bond geometry (Å, °)

*D*—H⋯*A*	*D*—H	H⋯*A*	*D*⋯*A*	*D*—H⋯*A*
N1—H1*N*⋯O1^i^	0.94 (4)	2.11 (4)	3.023 (4)	164 (3)
O2—H2*O*⋯N2	0.81 (4)	1.80 (4)	2.536 (4)	150 (4)

Symmetry code: (i) 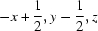.

**Fig. 1.**
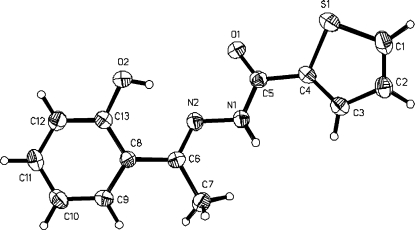
The molecular structure of the title compound showing 30% probability displacement ellipsoids and the atom-numbering scheme.

## supplementary crystallographic information

### Comment

Schiff-base have received considerable attention as they can be utilized as effective ligands in coordination chemistry. (Casas *et al.*, 2000). They are important intermediates which have been reported to be chiral coordination compounds with many interesting properties (Habermehl *et al.*, 2006). As part of our search for new schiff-base compounds we synthesized the title compound (I), and its crystal structure is determined herein.

The molecular structure of the title compound is shown in Fig. 1. In the molecule, the dihedral angle between the benzene ring and the thiophene ring is 10.07 (17)°. In the crystal structure, molecules are linked by the N—H···O hydrogen bonds to form one-dimensional chains along [010]. Bond lengths and angles agree with those common to related structures (Li & Jian, 2010*a*,*b*).

### Experimental

A mixture of 1-(2-hydroxyphenyl)ethanone (0.01 mol) and thiophene-2-carbohydrazide (0.01 mol) was stirred in refluxing ethanol (20 mL) for 2 h to afford the title compound (0.092 mol, yield 92%). Single crystals suitable for X-ray measurements were obtained by recrystallization of the title compound from ethanol at room temperature.

### Refinement

H atoms bonded to C atoms were fixed geometrically and allowed to ride on their attached atoms, with C—H = 0.93-0.96 Å, and *U*_iso_(H) = 1.2–1.5*U*_eq_(C). H atoms boned to N and O atoms were refined indpendently with isotropic displacement parameters.

### Crystal data

**Table:**

C_13_H_12_N_2_O_2_S	*F*(000) = 1088
*M**_r_* = 260.31	*D*_x_ = 1.386 Mg m^−^^3^
Orthorhombic, *P**b**c**a*	Mo *K*α radiation, λ = 0.71073 Å
Hall symbol: -P 2ac 2ab	Cell parameters from 2839 reflections
*a* = 13.454 (3) Å	θ = 3.0–27.5°
*b* = 7.6303 (15) Å	µ = 0.25 mm^−^^1^
*c* = 24.305 (5) Å	*T* = 293 K
*V* = 2495.1 (9) Å^3^	Block, colorless
*Z* = 8	0.25 × 0.20 × 0.19 mm

### Data collection

**Table:**

Bruker SMART CCD diffractometer	1047 reflections with *I* > 2σ(*I*)
Radiation source: fine-focus sealed tube	*R*_int_ = 0.156
graphite	θ_max_ = 25.0°, θ_min_ = 3.0°
φ and ω scans	*h* = −16→14
16044 measured reflections	*k* = −9→9
2189 independent reflections	*l* = −28→28

### Refinement

**Table:**

Refinement on *F*^2^	Primary atom site location: structure-invariant direct methods
Least-squares matrix: full	Secondary atom site location: difference Fourier map
*R*[*F*^2^ > 2σ(*F*^2^)] = 0.050	Hydrogen site location: inferred from neighbouring sites
*wR*(*F*^2^) = 0.126	H atoms treated by a mixture of independent and constrained refinement
*S* = 0.89	*w* = 1/[σ^2^(*F*_o_^2^) + (0.0573*P*)^2^] where *P* = (*F*_o_^2^ + 2*F*_c_^2^)/3
2189 reflections	(Δ/σ)_max_ < 0.001
172 parameters	Δρ_max_ = 0.23 e Å^−^^3^
0 restraints	Δρ_min_ = −0.29 e Å^−^^3^

### Special details

**Table:**

Geometry. All e.s.d.'s (except the e.s.d. in the dihedral angle between two l.s. planes) are estimated using the full covariance matrix. The cell e.s.d.'s are taken into account individually in the estimation of e.s.d.'s in distances, angles and torsion angles; correlations between e.s.d.'s in cell parameters are only used when they are defined by crystal symmetry. An approximate (isotropic) treatment of cell e.s.d.'s is used for estimating e.s.d.'s involving l.s. planes.
Refinement. Refinement of *F*^2^ against ALL reflections. The weighted *R*-factor *wR* and goodness of fit *S* are based on *F*^2^, conventional *R*-factors *R* are based on *F*, with *F* set to zero for negative *F*^2^. The threshold expression of *F*^2^ > σ(*F*^2^) is used only for calculating *R*-factors(gt) *etc*. and is not relevant to the choice of reflections for refinement. *R*-factors based on *F*^2^ are statistically about twice as large as those based on *F*, and *R*- factors based on ALL data will be even larger.

### Fractional atomic coordinates and isotropic or equivalent isotropic displacement parameters (Å^2^)

**Table:**

	*x*	*y*	*z*	*U*_iso_*/*U*_eq_	
S1	0.44225 (8)	0.02138 (15)	0.75337 (5)	0.0783 (4)	
O1	0.37088 (16)	0.2689 (3)	0.84114 (10)	0.0546 (7)	
O2	0.31584 (19)	0.3541 (4)	0.98652 (13)	0.0591 (8)	
N1	0.2282 (2)	0.1203 (4)	0.85835 (12)	0.0498 (8)	
N2	0.2212 (2)	0.2088 (4)	0.90867 (12)	0.0491 (8)	
C1	0.3999 (3)	−0.1057 (5)	0.70050 (16)	0.0698 (12)	
H1	0.4408	−0.1542	0.6737	0.084*	
C2	0.3020 (3)	−0.1273 (4)	0.70204 (15)	0.0562 (10)	
H2A	0.2668	−0.1921	0.6761	0.067*	
C3	0.2570 (3)	−0.0416 (4)	0.74723 (16)	0.0502 (9)	
H3A	0.1891	−0.0430	0.7543	0.060*	
C4	0.3251 (2)	0.0437 (4)	0.77919 (15)	0.0457 (9)	
C5	0.3114 (3)	0.1538 (5)	0.82858 (14)	0.0462 (9)	
C6	0.1375 (2)	0.2103 (4)	0.93416 (15)	0.0453 (9)	
C7	0.0439 (2)	0.1341 (5)	0.91119 (16)	0.0632 (11)	
H7A	0.0469	0.1355	0.8717	0.095*	
H7B	−0.0119	0.2024	0.9233	0.095*	
H7C	0.0366	0.0156	0.9238	0.095*	
C8	0.1386 (2)	0.2934 (4)	0.98860 (14)	0.0421 (9)	
C9	0.0523 (3)	0.3054 (4)	1.02020 (16)	0.0541 (10)	
H9A	−0.0066	0.2609	1.0059	0.065*	
C10	0.0510 (3)	0.3801 (5)	1.07136 (16)	0.0626 (11)	
H10A	−0.0080	0.3859	1.0912	0.075*	
C11	0.1374 (3)	0.4465 (5)	1.09333 (16)	0.0603 (11)	
H11A	0.1368	0.4981	1.1280	0.072*	
C12	0.2244 (3)	0.4366 (5)	1.06408 (16)	0.0571 (10)	
H12A	0.2827	0.4812	1.0791	0.069*	
C13	0.2261 (3)	0.3603 (4)	1.01196 (15)	0.0456 (9)	
H1N	0.186 (3)	0.023 (5)	0.8545 (16)	0.082 (13)*	
H2O	0.305 (3)	0.315 (6)	0.9561 (19)	0.094 (19)*	

### Atomic displacement parameters (Å^2^)

**Table:**

	*U*^11^	*U*^22^	*U*^33^	*U*^12^	*U*^13^	*U*^23^
S1	0.0552 (6)	0.0905 (8)	0.0893 (9)	−0.0020 (6)	0.0197 (6)	−0.0246 (7)
O1	0.0464 (15)	0.0593 (15)	0.0581 (17)	−0.0070 (12)	−0.0021 (12)	−0.0067 (13)
O2	0.0448 (16)	0.0713 (19)	0.061 (2)	−0.0090 (13)	0.0029 (15)	−0.0080 (16)
N1	0.0521 (19)	0.0515 (19)	0.046 (2)	−0.0051 (16)	0.0061 (15)	−0.0075 (17)
N2	0.0530 (19)	0.0485 (18)	0.046 (2)	−0.0035 (14)	0.0054 (15)	−0.0057 (16)
C1	0.085 (3)	0.063 (3)	0.061 (3)	0.003 (2)	0.019 (2)	−0.013 (2)
C2	0.074 (3)	0.044 (2)	0.051 (3)	0.006 (2)	0.004 (2)	−0.0002 (19)
C3	0.053 (2)	0.045 (2)	0.053 (2)	0.0025 (17)	0.008 (2)	0.004 (2)
C4	0.045 (2)	0.046 (2)	0.046 (2)	0.0064 (16)	0.0065 (17)	0.0029 (18)
C5	0.044 (2)	0.049 (2)	0.046 (2)	0.0054 (18)	−0.0015 (18)	0.0014 (19)
C6	0.042 (2)	0.045 (2)	0.050 (2)	−0.0035 (16)	−0.0012 (18)	0.0007 (18)
C7	0.054 (2)	0.068 (3)	0.068 (3)	−0.001 (2)	−0.003 (2)	−0.019 (2)
C8	0.043 (2)	0.044 (2)	0.038 (2)	−0.0019 (15)	0.0009 (17)	0.0021 (17)
C9	0.048 (2)	0.059 (3)	0.055 (3)	−0.0081 (18)	0.007 (2)	0.000 (2)
C10	0.062 (3)	0.075 (3)	0.051 (3)	0.000 (2)	0.015 (2)	0.005 (2)
C11	0.076 (3)	0.065 (3)	0.040 (2)	0.003 (2)	0.008 (2)	0.000 (2)
C12	0.062 (3)	0.056 (2)	0.053 (3)	−0.0039 (19)	−0.004 (2)	0.000 (2)
C13	0.047 (2)	0.041 (2)	0.049 (2)	0.0011 (17)	0.0033 (18)	0.0015 (19)

### Geometric parameters (Å, °)

**Table:**

S1—C4	1.705 (3)	C6—C8	1.467 (5)
S1—C1	1.708 (4)	C6—C7	1.495 (4)
O1—C5	1.227 (4)	C7—H7A	0.9600
O2—C13	1.357 (4)	C7—H7B	0.9600
O2—H2O	0.81 (4)	C7—H7C	0.9600
N1—C5	1.356 (4)	C8—C9	1.395 (4)
N1—N2	1.400 (4)	C8—C13	1.402 (4)
N1—H1N	0.94 (4)	C9—C10	1.368 (5)
N2—C6	1.285 (4)	C9—H9A	0.9300
C1—C2	1.327 (5)	C10—C11	1.377 (5)
C1—H1	0.9300	C10—H10A	0.9300
C2—C3	1.415 (5)	C11—C12	1.371 (5)
C2—H2A	0.9300	C11—H11A	0.9300
C3—C4	1.366 (5)	C12—C13	1.395 (5)
C3—H3A	0.9300	C12—H12A	0.9300
C4—C5	1.477 (5)		
			
C4—S1—C1	91.4 (2)	C6—C7—H7A	109.5
C13—O2—H2O	105 (3)	C6—C7—H7B	109.5
C5—N1—N2	115.5 (3)	H7A—C7—H7B	109.5
C5—N1—H1N	126 (2)	C6—C7—H7C	109.5
N2—N1—H1N	115 (2)	H7A—C7—H7C	109.5
C6—N2—N1	119.0 (3)	H7B—C7—H7C	109.5
C2—C1—S1	112.4 (3)	C9—C8—C13	116.8 (3)
C2—C1—H1	123.8	C9—C8—C6	121.1 (3)
S1—C1—H1	123.8	C13—C8—C6	122.1 (3)
C1—C2—C3	112.9 (4)	C10—C9—C8	122.6 (4)
C1—C2—H2A	123.6	C10—C9—H9A	118.7
C3—C2—H2A	123.6	C8—C9—H9A	118.7
C4—C3—C2	112.0 (3)	C9—C10—C11	119.7 (4)
C4—C3—H3A	124.0	C9—C10—H10A	120.2
C2—C3—H3A	124.0	C11—C10—H10A	120.2
C3—C4—C5	130.5 (3)	C12—C11—C10	120.0 (4)
C3—C4—S1	111.3 (3)	C12—C11—H11A	120.0
C5—C4—S1	118.1 (3)	C10—C11—H11A	120.0
O1—C5—N1	122.7 (3)	C11—C12—C13	120.5 (4)
O1—C5—C4	121.9 (3)	C11—C12—H12A	119.8
N1—C5—C4	115.4 (3)	C13—C12—H12A	119.8
N2—C6—C8	115.4 (3)	O2—C13—C12	116.3 (3)
N2—C6—C7	123.7 (3)	O2—C13—C8	123.3 (3)
C8—C6—C7	120.9 (3)	C12—C13—C8	120.4 (3)
			
C5—N1—N2—C6	167.0 (3)	N2—C6—C8—C9	179.2 (3)
C4—S1—C1—C2	0.9 (3)	C7—C6—C8—C9	−0.7 (5)
S1—C1—C2—C3	−0.5 (4)	N2—C6—C8—C13	−2.3 (5)
C1—C2—C3—C4	−0.3 (5)	C7—C6—C8—C13	177.7 (3)
C2—C3—C4—C5	177.6 (3)	C13—C8—C9—C10	0.7 (5)
C2—C3—C4—S1	1.0 (4)	C6—C8—C9—C10	179.2 (3)
C1—S1—C4—C3	−1.1 (3)	C8—C9—C10—C11	−0.1 (6)
C1—S1—C4—C5	−178.1 (3)	C9—C10—C11—C12	−0.4 (6)
N2—N1—C5—O1	−8.5 (5)	C10—C11—C12—C13	0.3 (6)
N2—N1—C5—C4	172.4 (3)	C11—C12—C13—O2	−179.1 (3)
C3—C4—C5—O1	−153.3 (4)	C11—C12—C13—C8	0.4 (5)
S1—C4—C5—O1	23.1 (4)	C9—C8—C13—O2	178.5 (3)
C3—C4—C5—N1	25.8 (5)	C6—C8—C13—O2	0.1 (5)
S1—C4—C5—N1	−157.8 (3)	C9—C8—C13—C12	−0.8 (5)
N1—N2—C6—C8	175.2 (3)	C6—C8—C13—C12	−179.3 (3)
N1—N2—C6—C7	−4.8 (5)		

### Hydrogen-bond geometry (Å, °)

**Table:**

*D*—H···*A*	*D*—H	H···*A*	*D*···*A*	*D*—H···*A*
N1—H1N···O1^i^	0.94 (4)	2.11 (4)	3.023 (4)	164 (3)
O2—H2O···N2	0.81 (4)	1.80 (4)	2.536 (4)	150 (4)

Symmetry codes: (i) −*x*+1/2, *y*−1/2, *z*.
